# Predicting circRNA-miRNA interactions utilizing transformer-based RNA sequential learning and high-order proximity preserved embedding

**DOI:** 10.1016/j.isci.2023.108592

**Published:** 2023-11-29

**Authors:** Jiren Zhou, Xinfei Wang, Rui Niu, Xuequn Shang, Jiayu Wen

**Affiliations:** 1 School of Computer Science, Northwestern Polytechnical University, Xi’an, China; 2 School of Information Engineering, Xijing University, Xi’an, China; 3 The John Curtin School of Medical Research, The Australian National University, Canberra, ACT 2601, Australia; 4 Australian Research Council Centre of Excellence for the Mathematical Analysis of Cellular Systems

**Keywords:** Molecular biology, Molecular network, Mathematical biosciences, Machine learning

## Abstract

A key regulatory mechanism involves circular RNA (circRNA) acting as a sponge to modulate microRNA (miRNA), and thus, studying their interaction has significant medical implications. In this field, there are currently two pressing issues that remain unresolved. Firstly, due to the scarcity of verified interactions, we require a minimal amount of samples for training. Secondly, the current models lack interpretability. Therefore, we propose SPBCMI, a method that combines sequence features extracted using the Bidirectional Encoder Representations from Transformer (BERT) model and structural features of biological molecule networks extracted through graph embedding to train a GBDT (Gradient-boosted decision trees) classifier for prediction. Our method yielded an AUC of 0.9143, which is currently the best for this problem. Furthermore, in the case study, SPBCMI accurately predicted 7 out of 10 circRNA-miRNA interactions. These results show that our method provides an innovative and high-performing approach to understanding the interaction between circRNA and miRNA.

## Highlights

• The BERT model can provide a more comprehensive understanding of RNA_sequence
• The BERT model can provide interpretability from a token-based perspective
• Models based on BERT exhibit strong generalization capabilities
• Combining NLP models and graph machine learning can improve model’s performance

## Graphical abstract

**Figure:**
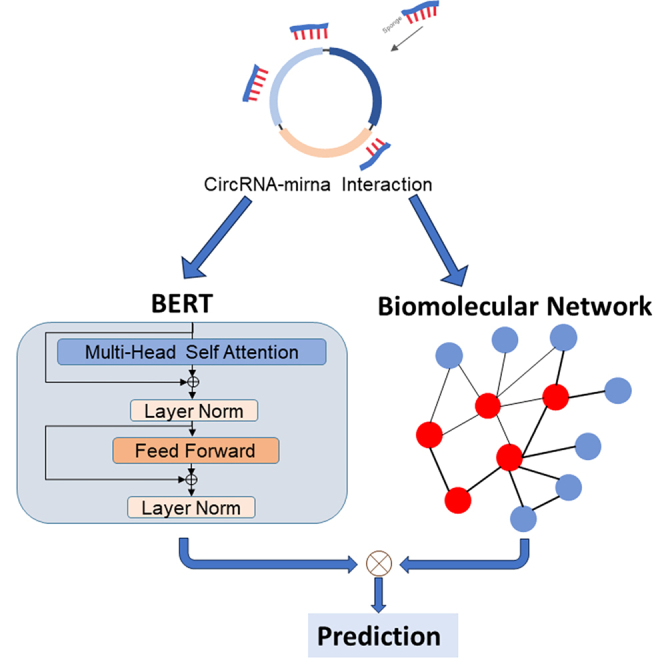

## Introduction

In the preceding decade, the advancement of RNA-sequencing (RNA-seq) methodologies and the development of specialized computational pipelines have culminated in the generation of substantial datasets related to non-coding RNA (ncRNA). This has facilitated the elevation of ncRNA to a prominent position within contemporary research paradigms. Consequently, it has engendered novel perspectives and promising avenues for the diagnosis and therapeutic intervention of human pathologies.

Circular RNA (circRNA), an innovative classification of ncRNA, is synthesized through the back-splicing process of mRNA precursors (pre-mRNAs). Divergent from conventional linear mRNAs, circRNAs are characterized by a covalently closed-loop structure, devoid of free ends. This imparts superior stability and conservation to circRNAs relative to their linear RNA counterparts. Research has elucidated that circRNAs encompass an abundance of microRNA (miRNA) response elements, thereby enabling them to interact with and effectively serve as molecular sponges for miRNAs.^1^ CircRNAs modulate gene expression as miRNA sponges, regulate transcription and alternative splicing, interact with RNA-binding proteins to influence RNA networks, and partake in the regulation of translation, thereby influencing protein synthesis and function.^2^^,^^3^^,^^4^^,^^5^^,^^6^

Investigation into circRNA-miRNA-mediated models is instrumental in elucidating the pathogenesis of human diseases, fostering targeted diagnostic, therapeutic, and prognostic strategies. It will further enhance our comprehension of disease pathology by unveiling potential circRNA-miRNA interactions (CMIs). Employing graph theory for the prediction of potential edges has emerged as a prevalent method in recent years for the inference of associative relationships. Within these graphs, there are primarily two categories of features that can be extracted: the inherent attribute features of the nodes and the features of the network structure.

Natural Language Processing (NLP) methods have demonstrated promising outcomes in diverse bioinformatics investigations by employing sequential feature extraction as the intrinsic attribute features of nodes. This entails employing a common strategy involving various machine learning techniques, including CNN (Convolutional neural network), RNN (Recurrent Neural Networks ), autoencoders, and others, for feature extraction after tokenization and conversion of sequence data through word2vec.^6^^,^^7^^,^^8^^,^^9^^,^^10^ Cao et al.^11^ proposed CircSSNN, a model inspired by Bidirectional Encoder Representations from Transformer (BERT). This innovative end-to-end model for predicting circRNA-binding sites, using a unique combination of static local context, dynamic global context information, and a network architecture known as Seq_Transformer. This architecture, bolstered by ResNet and LayerNorm modules, increases model robustness, reduces hyperparameter sensitivity, and provides better performance across multiple RNA-RBP combination recognition tasks.

In the prediction of association relationships, numerous models have been proposed for the extraction of network structure features. Ji et al.^12^ employed Learning Graph Representations with Global Structural Information (GraRep) extracting the structure of a heterogeneous network constructed by several molecules. HGANMDA, a hierarchical graph attention network model, was developed by Li and their team for predicting miRNA-disease associations, which integrates multiple datasets and leverages node-layer and semantic-layer attention for feature aggregation and semantic information extraction.^13^ Zheng et al.^14^ proposed an advanced miRNA-disease association prediction model, named iMDA-BN. This model utilizes biological networks and graph embedding algorithms, designed to represent miRNA and disease characteristics from a holistic network perspective, thus facilitating predictions for new disease-miRNA pairs. Li et al.^15^ put forward SDNE-MDA, in which the structural deep network embedding (SDNE) was introduced to extract features from the heterogeneous molecular associations network.

Research surrounding CMIs have, in recent years, progressively emerged as a frontier issue in conjunction with the accumulation of biological experimental data. Correspondingly, the computational methodologies applied in this domain have experienced a substantial upsurge. Within this growing field, certain model extract structure feature of network for predictions. For instance, NECMA, a computational method proposed by Lan et al. which based on network embedding and GCNCMI which was put forward by He et al. via introducing the graph convolutional neural network-based approach^16^^,^^17^ both leverage structural features to improve the performance. Enhancing feature sets with molecular sequence traits and similarity kernels can bolster prediction accuracy in computational models. Specifically, Models like CMASG (a computational model using a graph neural network and singular value decomposition),^18^ WSCD (Word2vec, SDNE, Convolutional Neural Network and Deep Neural Network),^19^ and IIMCCMA^20^ utilize diverse methodologies, including Gaussian interaction kernels, variational autoencoders, word2vec, network embeddings, and inductive matrix completion, to enhance the precision of CMIs predictions. However, reducing computational complexity and eliminating noise are crucial aspects for model performance. Models like KGDCMI^21^ and SGCNCMI^22^ navigate the challenges of high-dimensional and potentially noisy data by utilizing machine learning techniques such as the k-m r algorithm, the high-order proximity preserved embedding (HOPE) algorithm,^23^ and sparse autoencoders. These models effectively distill useful information from complex molecular attributes and behavioral features, improving the accuracy of CMIs predictions.

Existing models have made strides in feature extraction and multi-dimensional prediction, yet challenges persist. (1) A key challenge is few-shot. Validated CMIs, as confirmed through biological experiments, are significantly sparse in comparison to their potential quantity. In the dataset we utilized, we had only 2,346 circRNAs and 962 miRNAs. In exoRBase 2.0,^24^ there are already records of 79,084 circRNAs, and currently, there are approximately over 1,000 known miRNAs. The 9,905 pairs of relationships we used represent only an exceedingly small fraction in comparison to the potential number of interactions. The challenge is that we need to consider a more robust algorithm that can generalize the predicting tasks with fewer labeled data. (2) The “black box.” Another issue is the limited interpretability of existing algorithms. One distinctive characteristic of biological sequences is that, when treating the k-mer processed sequences as sentences and k-mer fragments as tokens, the interactions between tokens are not linear or locally convoluted. Instead, a token’s interaction with the current token is significantly influenced by tokens that are far away in the preceding and following context. This implies that the secondary structure of RNA can have a substantial impact on binding.

**Figure 1:**
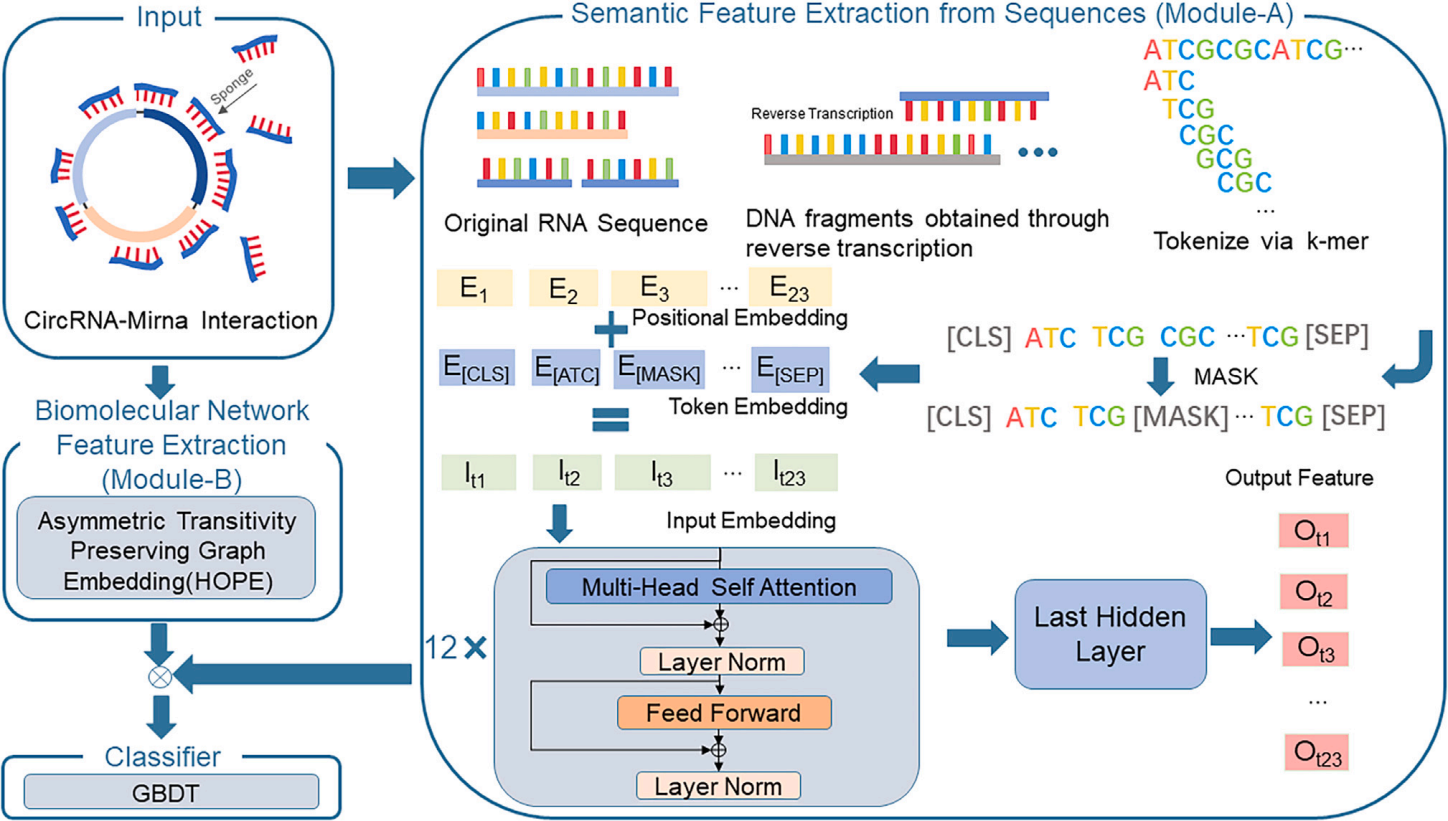
Flowchart depicting the complete experimental workflow This figure presents a comprehensive workflow of our data processing and analysis methodology. The input data are categorized into two distinct types—RNA_sequence feature and biological network structure feature. The RNA_sequence data undergoes several stages of feature extraction through module-A. Firstly, k-mer analysis is employed to divide the sequence into subsequences of length “k.” Then, a fine-tuned BERT model, specifically tailored for biological sequences, is used to extract high-level semantic features from the sequences. Parallelly, the biological network structure feature is extracted using the HOPE (high-order proximity preserved embedding) algorithm in module-B. This method generates a low-dimensional vector representation of the complex network. Subsequently, the features obtained from both sources are concatenated to form a composite feature matrix. This matrix is then fed into GBDT for training.

In order to address the limitations of existing models, we introduce the model, SPBCMI (model for predicting potential CMI interactions via Sequence Processed by Bidirectional Encoder Representations from Transformer). To address these issues, we utilized BERT, a language model trained on DNA data, for feature extraction. Here, we leveraged two known characteristics of the BERT model to tackle the two problems we identified. Firstly, models trained on larger datasets and then fine-tuned typically outperform those trained on smaller datasets. The pretrained model we used to be currently the largest in terms of training data scale. Furthermore, given the low conservation of circRNAs and the continual increase in their numbers, using pre-transcribed sequence data for training ensures that the model retains its feature extraction capabilities when faced with new sequencing data in the future. Secondly, BERT’s architecture is bidirectional, allowing it to compute attention matrices between tokens. This means that it simultaneously extracts the influence of tokens in the same sequence, both before and after the current token. This reflects the reality of sequences, where the secondary structure of a token on the same sequence can be determined by the surrounding sequences in both directions. Moreover, different circRNAs with variations in their sequences might express the same biological semantics due to connections between tokens at various distances. Therefore, using BERT is better suited to capture these situations. Simultaneously, we used structural information from biological molecular networks to supplement and enhance the accuracy of our algorithm. The detailed procedure is outlined in Figure 1. Initially, all RNA sequences are reverse transcribed into DNA sequences. Subsequently, sequence feature extraction is performed by employing a BERT model trained on a comprehensive genome-wide DNA dataset. We then utilize HOPE to extract structural features. Ultimately, these two types of features are concatenated and fed into a GBDT^25^ classifier for training and predicting circRNA-miRNA associations. Under the 5-fold cross-validation, SPBCMI achieves the area under the curve (AUC) of 0.9100 and the area under the precision-recall curve (AUPR) of 0.8900, results that are, to the best of our knowledge, unrivalled. In a case study, seven out of the top ten highest probability results generated by SPBCMI are corroborated by findings in PubMed. These outcomes illustrate that our model delivers reliable and exceptional performance in the task of predicting circRNA-miRNA associations.

## Results

### Test set splitting and evaluation metrics

When assessing the final performance of our model, we employed a 5-fold cross-validation approach. This means that we randomly shuffled the entire dataset and divided it into five equal parts. In each iteration, one part was used as the test set, while the remaining four parts constituted the training set. Additionally, for other comparative experiments, we randomly shuffled the entire dataset and allocated 20% as the test set, with the remaining 80% serving as the training set.

### Evaluation of model performance via 5-fold cross-validation

**Figure 2:**
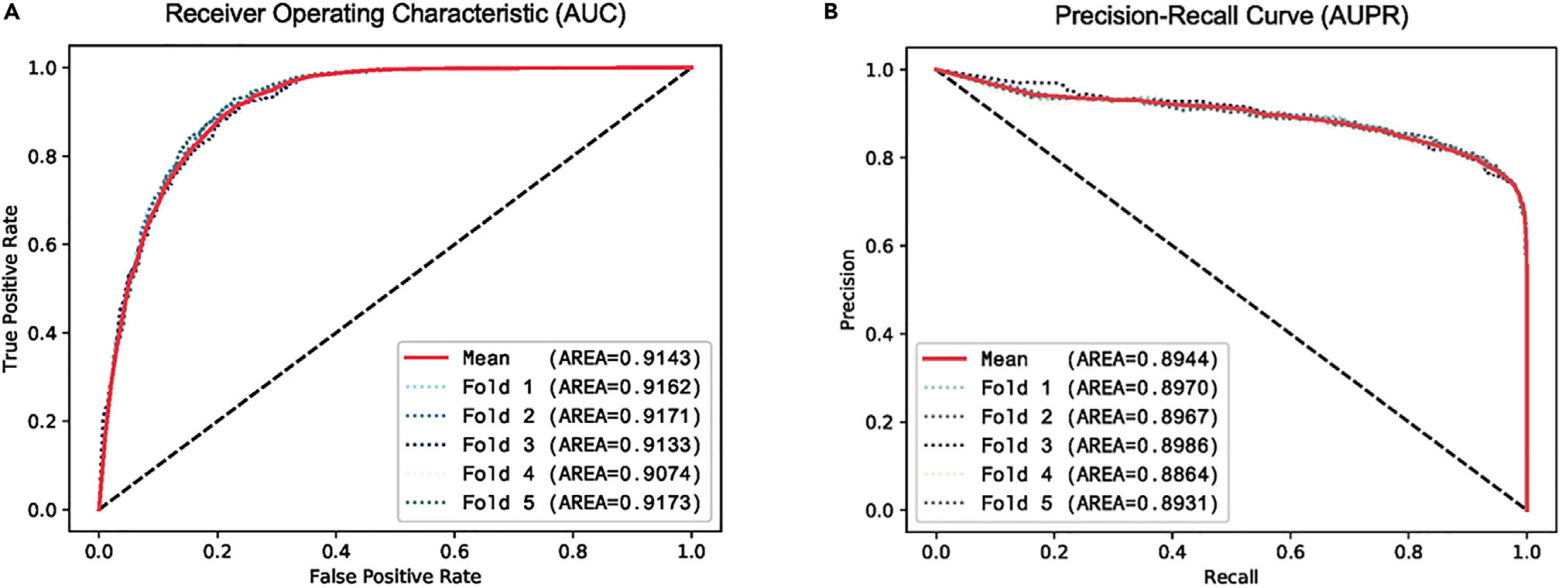
5-Fold cross-validation results (A) The ROC of 5-folds and mean results. (B) The PRC of 5-folds and mean results. The average AUC achieved by our model was 0.9143(Variance: 1.381E-05), indicating an elevated level of discriminative ability in distinguishing between positive and negative samples. Additionally, the average AUPR reached 0.8944(Variance: 1.907E-05), highlighting the model’s effectiveness in capturing the trade-off between precision and recall. The results consistently demonstrated that the model’s performance curves remained above the diagonal line, indicating a smooth and effective classification.

We implemented a 5-fold cross-validation approach to assess the ultimate performance of our model. Graphical representations of the ROC (Receiver Operating Characteristic) and the PRC (Precision-Recall Curve) are depicted in Figure 2. A comprehensive tabulation of all evaluation metrics can be found in Table 1.

**Table 1:** Performance evaluation of SPBMCI via 5-fold cross-validation

Fold	MCC	ACC	F1
1	0.6860	0.8400	0.8506
2	0.6990	0.8460	0.8552
3	0.6783	0.8364	0.8477
4	0.6693	0.8329	0.8436
5	0.6993	0.8470	0.8540
Mean	0.6864	0.8405	0.8502
Variance	1.367E-04	2.946E-05	1.788E-05

Table 1 summarizes the performance metrics, including MCC, ACC, AUC, AUPR, and F1, obtained from the five-fold cross-validation. The average AUC reached 0.9143. Additionally, AUPR, ACC, MCC, and F1 reached 0.8944, 0.8405, 0.6864, and 0.8502, respectively.

The evaluation of the classification model using 5-fold cross-validation demonstrates its robustness and efficiency in accurately predicting the target variables. The performance metrics MCC (Matthews Correlation Coefficient), ACC (Accuracy), AUC, AUPR, and F1 provide quantitative evidence of the model’s effectiveness. The consistently high values and the position of the performance curves above the diagonal line indicate a smooth and reliable classification performance. These findings validate the efficacy of our classification model for the given task.

### Comparative analysis of different k values in word segmentation

The performance of NLP models can be influenced by word segmentation, a crucial step in NLP. In our study, we aimed to investigate the impact of different word segmentation approaches on the performance. While keeping the other variables constant, we sequentially fine-tuned the DNABERT model using k values ranging from 3 to 6 and conducted comparative experiments. Then we observed that the model achieved stable and optimal results when utilizing a k value of 3.

**Figure 3:**
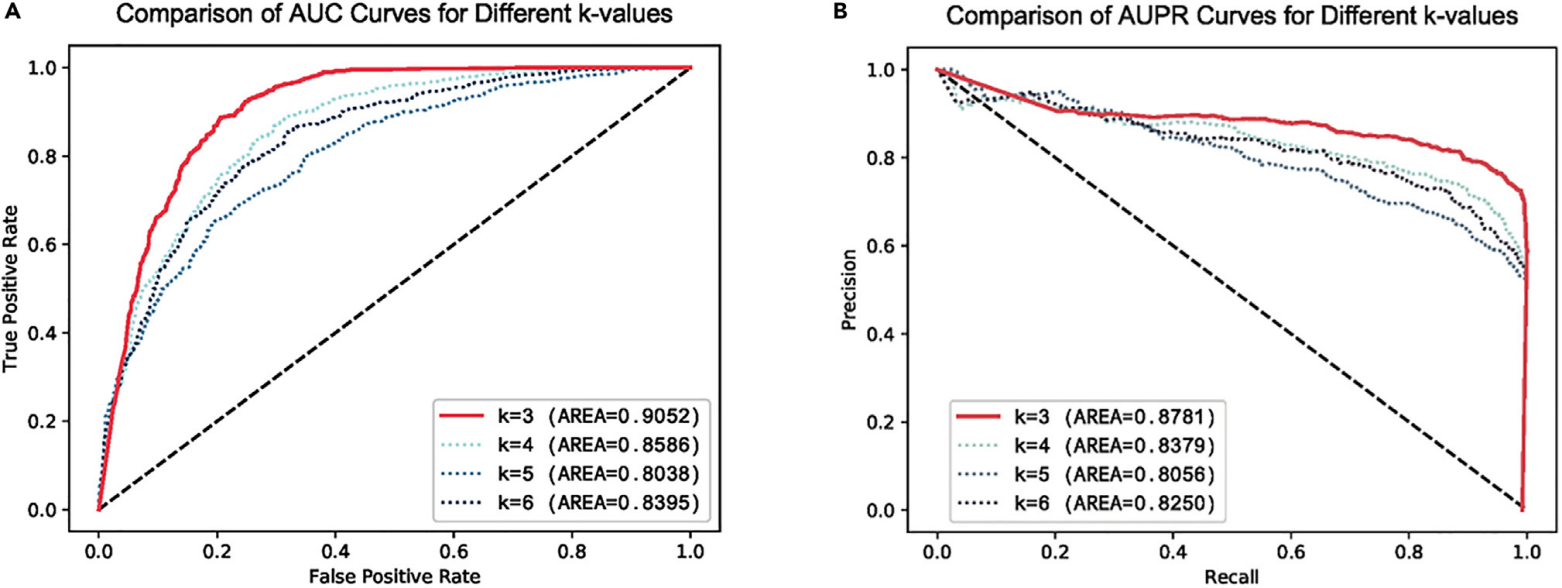
Comparative plots of the ROC and the PRC for different k values (A) ROC of different k-values. (B) PRC of different k-values. In the evaluation of word segmentation, we observed significant improvements in performance when using a k value of 3. The AUC and AUPR values reached 0.9052 and 0.8781, respectively, for k = 3. These values were noticeably higher than those obtained for other k values. Additionally, the curves of k = 3 exhibited smoother patterns, indicating superior performance compared to other k values.

The comparative plots of the ROC and the PRC are shown in Figure 3, and the comparative metrics are shown in Table 2.

**Table 2:** Performance evaluation of word segmentation

k value	MCC	ACC	F1
k = 3	0.6697	0.8324	0.8428
k = 4	0.5740	0.7857	0.7965
k = 5	0.4437	0.7216	0.7170
k = 6	0.5245	0.7600	0.7760

Table 2 presents the comparative results of word segmentation using different k values. The results show that the model achieved the best performance when using a word segmentation value of k = 3. ACC, MCC, and F1 reached 0.8324, 0.6697, and 0.8428, respectively.

### Comparative analysis of different sequence feature extraction methods

As a module that extracts sequence features through NLP methods, we compared module-A with other commonly used text feature extraction methods, including CNN, RNN, and autoencoder, in previous studies. We began by processing the sequences using k-mer with k = 3, and then encoded sequence into 128-dimensional vectors using Word2Vec. Subsequently, we fed these 128-dimensional vectors into CNN, RNN, and autoencoder for feature extraction. For CNN, we used convolutional layers with a kernel size of 3, a stride of 1, three convolutional layers, followed by flattening and fully connected layers to obtain a 64-dimensional vector. The RNN had a hidden layer size of 32. The learning rates for all three models were set to 0.001, and training continued until the models converged. The utilization of the BERT-based model resulted in improved performance due to its ability to capture contextual information and semantic relationships within the text. The model’s representation learning capabilities contributed to more meaningful and informative feature vectors.

**Figure 4:**
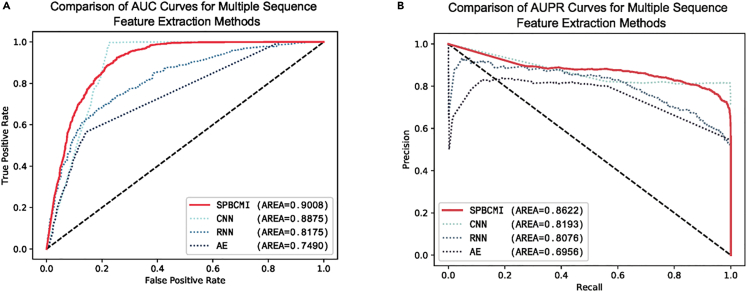
Comparative performance analysis of feature extraction methods on the ROC and the PRC (A) ROC of different method. (B)PRC of different method. The ROC and PRC demonstrate the performance of different methods, including autoencoders, CNN, RNN, and our model. The BERT-based model achieves an AUC of 0.9008 and an AUPR of 0.8622, indicating its superior performance. Notably, the curves for AUC and AUPR exhibit smoother patterns with fewer fluctuations, further validating the effectiveness and stability of the BERT-based model.

The plots of AUC and AUPR were shown in Figure 4. The metrics was shown in Table 3.

**Table 3:** Performance evaluation of sequence feature extraction

Method	MCC	ACC	F1
SPBCMI	0.6723	0.8324	0.8448
CNN	0.7710	0.8776	0.8892
RNN	0.4822	0.7410	0.7399
Autoencoder	0.3947	0.6782	0.5936

Table 3 presents that SPBCMI outperformed other methods, achieving the best performance. Specifically, the AUC score reached 0.9008, while AUPR, Acc., MCC, and F1 reached 0.8622, 0.8324, 0.6723, and 0.8448, respectively.

The experimental findings suggest that the BERT-based model, as a feature extractor in NLP, demonstrates superior effectiveness in extracting meaningful information from sequence data compared to other methods. As a method for generating word vectors, the BERT model leverages a bidirectional Transformer mechanism. Specifically designed for segmented sequence features, this approach effectively captures potential textual information associations within the arrangement of nucleotides. Consequently, the BERT model exhibits enhanced interpretability, enabling researchers to gain deeper insights into the underlying patterns and relationships within the sequence data.

### Comparative analysis of network embedding methods for extracting biological information networks

The information derived from constructing biological molecular networks plays a crucial role in improving the accuracy and fault tolerance of models. As a supplementary source of information, it enhances the performance of models. In this section, we employed network embedding methods to extract information from biological molecular networks. We compared module-B of SPBCMI with various widely used graph embedding methods in heterogeneous graphs.

The final test results demonstrated that HOPE outperformed other methods. HOPE exhibited significant advantages across various evaluation metrics.

**Figure 5:**
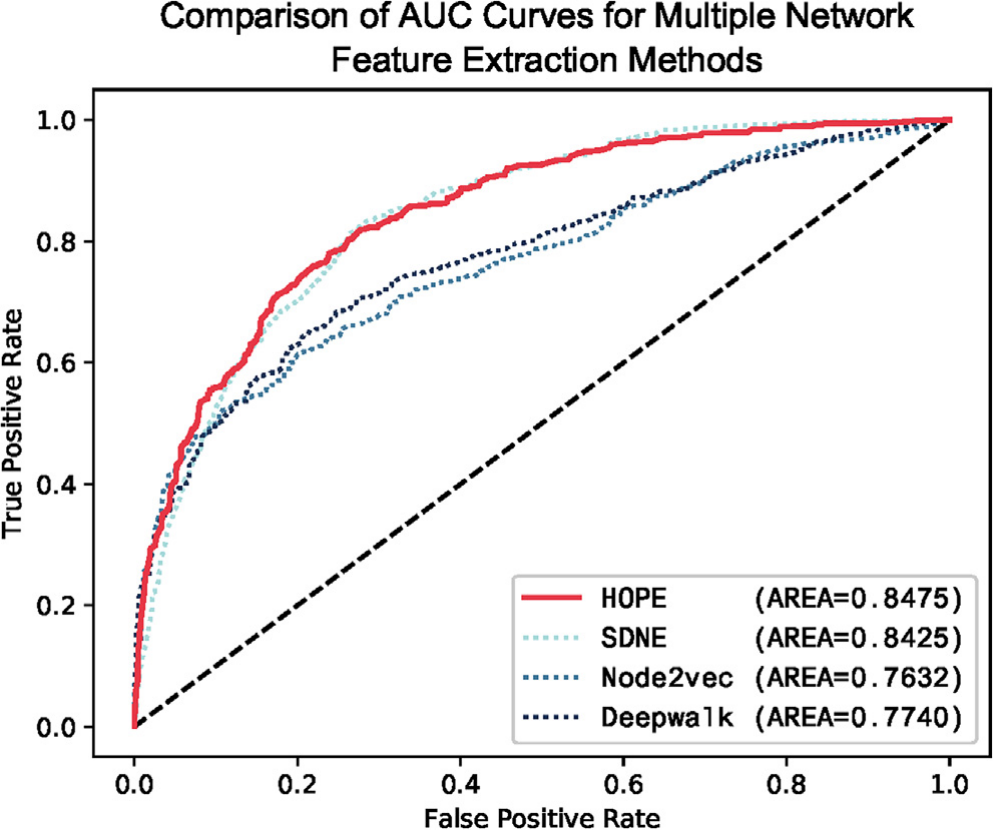
Comparison of AUC curves for multiple network feature extraction methods The AUC curve displays the performance of various network embedding methods, including HOPE and SDNE. HOPE exhibits the highest AUC value. The curve also illustrates that HOPE performs significantly better than other methods. Furthermore, the curve highlights the small gap between HOPE and SDNE. HOPE demonstrates a slight advantage over SDNE in terms of AUC and AUPR metrics.

In this regard, we plotted the AUC curve in Figure 5. Additionally, Table 4 presents the performance metrics.

**Table 4:** Performance evaluation of network feature extraction

Method	MCC	ACC	AUPR	F1
HOPE	0.5191	0.7826	0.8261	0.7412
SDNE	0.5664	0.7571	0.8258	0.7903
Node2vec	0.3739	0.6852	0.8017	0.6652
Deepwalk	0.3796	0.6890	0.8041	0.6771

The results from the comparative analysis of multiple network embedding methods revealed that HOPE achieved the best overall performance. Specifically, HOPE demonstrated the highest AUC score of 0.8475, surpassing other methods. Similarly, HOPE achieved an AUPR score of 0.8261, indicating its strong precision-recall performance.

The results from the comparative analysis of multiple network embedding methods revealed that HOPE achieved the best overall performance. Specifically, HOPE demonstrated the highest AUC score of 0.8475, surpassing other methods. Similarly, HOPE achieved an AUPR score of 0.8261, indicating its strong precision-recall performance.

### Comparative analysis of using individual features and combinations

**Figure 6:**
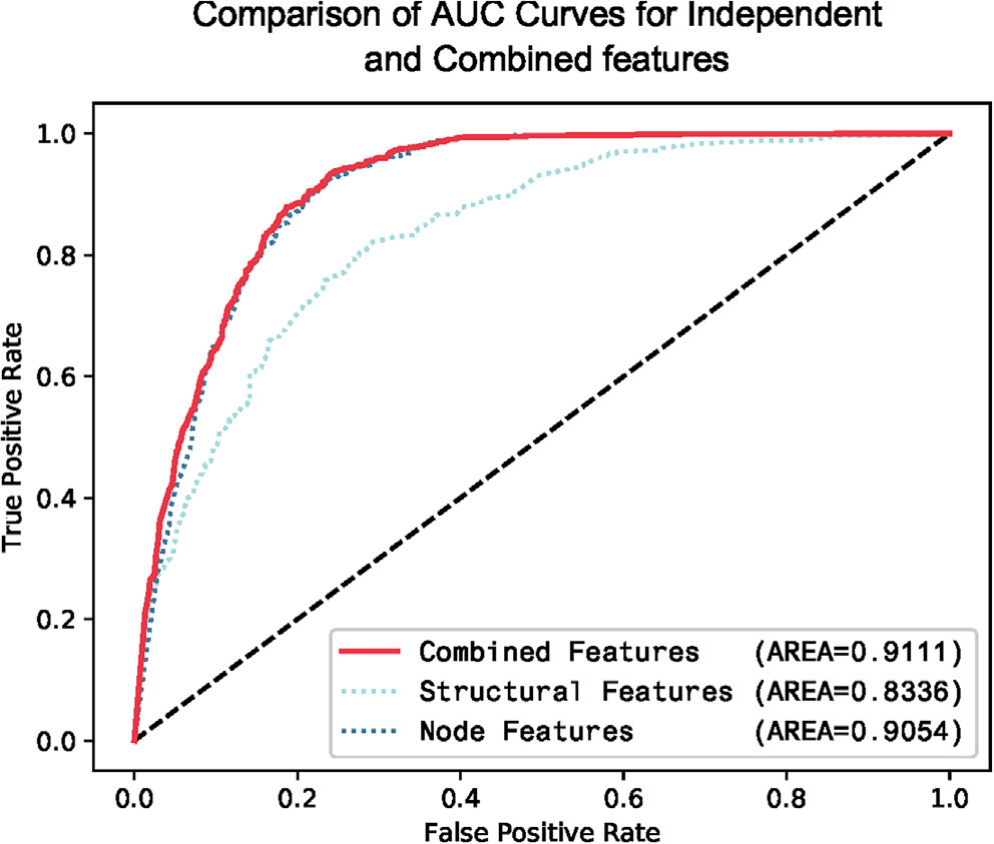
Comparative analysis of ROC for isolated features and integrated features The ROC depict the performance of various feature extraction methods, including node-based attributes, network-based attributes, and the combined features. The results demonstrate that the combined features yield the best performance, with an AUC of 0. 9111. These findings suggest that the integration of multiple feature sources is highly effective in enhancing the classification performance.

In this study, after extracting all the features, we compared the performance of the model using individual features separately for the classification task and the performance of the model after merging two types of features. The purpose was to demonstrate that utilizing multiple sources of features to complement each other can effectively enhance the predictive capability of the model without introducing additional noise. The results revealed that the integration of multiple features outperformed the use of individual features alone, indicating the efficacy of leveraging diverse feature sources for improved performance. The detailed results were shown in Figure 6 and Table 5.

**Table 5:** Performance evaluation of isolated features and integrated features

Feature	MCC	ACC	AUPR	F1
Combined Features	0.6905	0.8425	0.8888	0.8525
Structural Features	0.4975	0.7561	0.8195	0.7278
Node Features	0.6896	0.8417	0.8802	0.8522

The results from the comparative analysis of using isolated features and combined features demonstrated that the combined feature approach outperformed using individual features alone. The combined features achieved superior performance in terms of AUPR (0.8888), MCC (0.6905), ACC (0.8425), and F1 (0.8525). These scores were significantly higher than those obtained when using only the isolated features. This suggests that the integration of multiple features leads to improved performance across various evaluation metrics.

The results from the comparative analysis of using isolated features and combined features demonstrated that the combined feature approach outperformed using individual features alone. The combined features achieved superior performance in terms of AUPR (0.8888), MCC (0.6905), ACC (0.8425), and F1 (0.8525). These scores were significantly higher than those obtained when using only the isolated features. This suggests that the integration of multiple features leads to improved performance across various evaluation metrics.

### Comparative analysis: Demonstrating superior performance of our proposed method in CMI research

**Figure 7:**
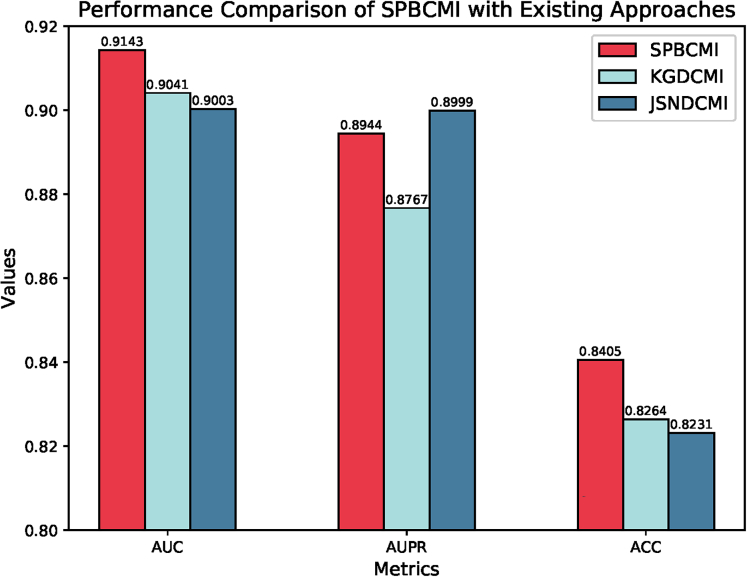
Performance comparison of AUC, AUPR, and ACC: Demonstrating the superiority of SPBCMI The figure presents a comprehensive comparison of three performance metrics: AUC, AUPR, and ACC, between our proposed method and other existing approaches. The results clearly demonstrate the significant advantages of our method in terms of AUC and AUPR, indicating its effectiveness in capturing the underlying patterns in the data.

In this study, we conducted a comparative analysis of our proposed method with existing approaches in the field of CMI research. To ensure fair comparisons, we followed the same data preprocessing methods and utilized the same dataset as the previous studies. By examining the reported results and extensively reviewing all available methods, we found that our approach outperformed the existing methods, achieving a state-of-the-art performance in terms of accuracy and efficiency. These findings suggest that our method has reached a local optimum in the field and offers improved outcomes for CMI analysis. A comprehensive comparison on AUC, AUPR, and ACC was shown in Figure 7. Furthermore, the metrics including AUC and AUPR for evaluation were shown in Table 6.

**Table 6:** Performance comparison of SPBCMI with existing model on AUC and AUPR

Metrics	SPBCMI	KGDCMI	WSCD	SGCNCMI	JSNDCMI
AUC	0.9143	0.8930	0.8923	0.8942	0.9003
AUPR	0.8944	0.8767	0.8935	0.8887	0.8999

Table 6 provides a comprehensive comparison of five different methods in terms of their AUC and AUPR. Our method achieved an impressive AUC score of 0.9143, reaching a local optimum in performance. Additionally, our method also exhibited a notable advantage in terms of AUPR, further demonstrating its effectiveness in capturing relevant information.

The table provides a comprehensive comparison of five different methods in terms of their AUC and AUPR. Our method achieved an impressive AUC score of 0.9143, reaching a local optimum in performance. Additionally, our method also exhibited a notable advantage in terms of AUPR, further demonstrating its effectiveness in capturing relevant information.

Based on these results, we conclude that our SPBCMI method outperforms the existing approaches. Based on the above analysis, it can be concluded that the SPBCMI method outperforms the existing approaches in terms of prediction accuracy and effectiveness.

### Case study

In order to validate the effectiveness of our algorithm, a case study was conducted using known information to train the model. The model was then used to predict the outcomes for unknown relationships, which were subsequently validated. Specifically, we examined the top ten ranked results, and remarkably, seven of them were confirmed through validation. This compelling result highlights the robustness of our method in effectively extracting valuable information and providing reliable insights for biological experiments. The ability of our algorithm to accurately predict previously unknown relationships demonstrates its potential to contribute significantly to the field, offering valuable guidance for future biological research endeavors. The detailed results of the case study are recorded in Table 7.

**Table 7:** Top 10 CMIs with the highest score predicted by SPBCMI

Ranking	circRNA	miRNA	Status
1	hsa_circ_0041103	hsa-miR-103a-3p	PMID:27484176
2	hsa_circ_0000554	hsa-miR-339-5p	PMID:27465405
3	hsa_circ_0041089	hsa-miR-3192-5p	unconfirmed
4	hsa_circ_0092306	hsa-miR-197-3p	PMID:31689616
5	hsa_circ_0007915	hsa-miR-106a-3p	PMID:28727484
6	hsa_circ_0002142	hsa-miR-625-5p	PMID:30988674
7	hsa_circ_0013870	hsa-miR-370-3p	unconfirmed
8	hsa_circ_100242	hsa-miR-145-5p	PMID:32218853
9	hsa_circ_0000977	hsa-miR-874-3p	PMID:29454093
10	hsa_circ_0005461	hsa-miR-4672	unconfirmed

## Discussion

The role of CMI in the regulatory dynamics of various cancers is becoming increasingly apparent, thereby necessitating a more profound understanding of these relationships. Computational methodologies provide an economical means of conducting such investigations, offering guidance for subsequent biological experiments. Applying different NLP methods to k-mer processed sequences is a common approach in sequence analysis. However, BERT stands out as a language model that is well-suited to RNA sequences because it considers the influence of a token from both preceding and following tokens. This approach potentially identifies the impact of secondary structures on token representations.

To this end, we introduced SPBCMI, the method that integrates techniques from NLP and network embedding. Leveraging the capabilities of the fine-tuned DNABERT model, SPBCMI captures sequence features, which are then supplemented with features obtained from graph embedding via the HOPE algorithm. By taking advantage of bidirectional multi-head attention mechanism, SPBCMI can effectively extend the small sample size to the scope of the whole genome from the perspective of word vectors. This approach is more interpretable compared to unsupervised methods, and more comprehensively captures information.

Following a series of comparative experiments, SPBCMI demonstrated superior performance over previous methodologies. Moreover, in a case study, it accurately predicted 7 out of 10 correct interaction pairs, further highlighting the efficacy of our method in predicting CMIs.

However, despite the contributions of SPBCMI, some limitations warrant attention. For instance, the negative samples used in this study were entirely randomly generated due to a lack of verified negative samples. In future work, we aim to develop strategies for generating negative samples based on a smaller number of examples. We believe that addressing these challenges will further refine our understanding of CMIs and enhance the predictability of computational models in this domain.

### Limitations of the study

Our study still presents opportunities for improvement. Firstly, BERT can only accept up to 512 tokens as input at a time, limiting our ability to extract features from long sequences of up to 70,000 bp in circRNAs. We intend to explore improvements in the encoding layer in future work to enable our model to accept a greater number of tokens in a single input. Secondly, our model’s tokenization using the k-mer method may not fully align with biological semantics. In future work, we aim to enhance the tokenization approach by considering statistical or data compression-based methods.

## STAR★Methods

### Key resources table

**Table:** 

REAGENT or RESOURCE	SOURCE	IDENTIFIER
**Deposited data**		

CMI-9905	KGDCMI	https://github.com/1axin/KGDCMI

**Other**		

Materials	This manuscript	https://github.com/ntitatip/SPBCMI
Data and code	This manuscript	https://github.com/ntitatip/SPBCMI

### Resource availability

#### Lead contact

Further information and requests for raw data and code should be directed to and will be fulfilled by the lead contact, JIAYU WEN (jiayu.wen@anu.edu.au).

#### Materials availability

All materials reported in this paper will be shared by the lead contact upon request.

#### Data and code availability

• RNA-seq data have been deposited at GitHub and are publicly available as of the date of publication. Accession numbers are listed in the key resources table.
• All original code has been deposited at GitHub and is publicly available as of the date of publication. Accession numbers are listed in the key resources table.
• Any additional information required to reanalyze the data reported in this paper is available from the lead contact upon request.

### Experimental model and study participant details

#### Dataset

In order to effectively evaluate the performance of our model, we adopted the same dataset as described in the KDGCMI I.^21^ Following the dataset description provided in their paper, we collected data from CircBank^26^ and CirR2Cancer.^27^ The data are represented in two separate files. One file contains all the node information, where each node represents an RNA molecule, consisting of 2346 circRNAs and 962 miRNAs, totaling 3308 nodes. The other file contains 9905 relationships formed by these two types of molecules, with circRNAs on the left and miRNAs on the right. We concatenate the embedded biological molecular network features and extraction of the sequence features as our positive dataset.

#### Methods for generating negative samples and composing training dataset

Because the experiments can only provide us with clear positive samples with validated relationships between the two types of RNA, we need to generate negative samples to form the training dataset. The generation method is as follows: Assuming in n round, we randomly select one circRNA and one miRNA, ensuring that this relationship does not overlap with the positive samples or with the previously generated n−1 negative samples. We continue this process until we have generated 9905 negative samples, which is the same number as the positive samples. This method is quite common and aligns with probability distributions, as the probability of two RNA molecules having no interaction is significantly higher than the probability of having relationship. In each experiment, we generate new negative samples randomly. We save the newly constructed dataset for each experiment to ensure reproducibility.

#### Methods for processing sequence data

Before extracting features using the BERT module, sequence data undergo the following processing steps. BERT has a maximum sequence length limit of 512 tokens, and each sequence begins with “[CLS]” and ends with “[SEP],” leaving 510 tokens for content. For cases where the sequence has fewer than 510 tokens, we pad it with the “[PAD]” symbol to reach the 510-token limit. For sequences longer than 510 tokens, we truncate them in 510-token segments and split them into multiple sequences. After extracting, these segments are concatenated together. This aligns with the data processing guidelines described in pre-trained models.

#### Fine-tuned Bidirectional Encoder Representations from Transformer model

In this study, we fine-tuned the pretrained DNABERT model which was repurposed to represent RNA sequence. To provide some background, DNABERT was originally pretrained by Ji et al.^28^ employing k-mer (where k ranges from 3 to 6) representations of nucleotide sequences derived from a human reference genome, GRCh38.p13.

DNABERT initially accepts a collection of sequences, which are presented as k-mer tokens, as input. Each sequence is characterized as a matrix M by transforming each token into a numerical vector. In essence, DNABERT assimilates contextual information by executing the multi-head self-attention mechanism on matrix M.

(Equation 1) $$\begin{array}{c} Multi\left(M\right)=\text{Concat}\left({\text{head}}_{1},\ldots ,{\text{head}}_{h}\right)W^{O} \end{array}$$

where,

(Equation 2) $$\begin{array}{c} head_{i}=\text{softmax}\left(\frac{MW_{i}^{Q}MW_{i}^{K^{T}}}{\sqrt{d_{k}}}\right)\cdot MW_{i}^{V} \end{array}$$

The linear projection parameters, $W_{i}^{Q},W_{i}^{K},W_{i}^{V}$ for each head $h_{i}=0$, are learned and used in the subsequent process. The next hidden states of M are determined by head, which initially calculates attention scores between pairs of tokens, and then employs these scores as weights to aggregate the rows of $MW_{i}^{V}$. In MultiHead(), the outputs of h independent heads, each with a distinct set of $W_{i}^{Q},W_{i}^{K},W_{i}^{V}$, are concatenated. This entire operation is executed L times, corresponding to the number of layers, L.

Through a self-supervised approach, DNABERT is trained to understand the fundamental syntax and semantics of DNA, using sequences of lengths ranging from 10 to 510, derived from the human genome through truncation and sampling. Each sequence contains randomly masked regions composed of k contiguous tokens, representing 15% of the total sequence. This enables DNABERT to predict the masked sequences based on the remaining portion, providing abundant training examples. Cross-entropy loss, denoted as $L=\sum_{i=0}^{N}-y_{i}^{\prime }\log\left(y_{i}\right)$, is used to pre-train DNABERT. In this formulation, $y_{i}^{\prime }$ and $y_{i}$ represent the ground truth and the predicted probability, respectively, for each class out of N total classes. For every subsequent application, the process was initiated with the pre-trained parameters, and DNABERT was then fine-tuned utilizing data specific to the task at hand. The optimizer used was AdamW, incorporated with a fixed weight decay. Additionally, dropout was applied to the output layer to improve the model’s robustness and to prevent overfitting. The number of epochs we set for fine-tuning is 3. We utilized the features extracted from BERT by setting the last hidden layer to be 64 dimensions.

#### The structural features of the biological network

The biomolecular network composed of circRNA-miRNA nodes constitutes a heterogeneous graph. Utilizing features obtained through graph embedding can enhance the accuracy of the model. In this context, we employed HOPE^23^ to capture the structural characteristics of the biological network.

Firstly, we provide a mathematical definition of graph embedding, followed by a detailed exposition of the HOPE method. A graph, denoted as G(V,E), consists of a set of vertices or nodes $V=\left\{v_{1},\ldots ,v_{n}\right\}$ , alongside an edge set $E={\left\{e_{i,j}\right\}}_{i,j=1}^{n}$. The objective of graph embedding is to discover a mapping function, such that $f:v_{i}\to x_{i}$ resides in $R^{d}$, where d is significantly less than the cardinality of V. The embedded vector, represented as $X_{i}=\left\{x_{1},x_{2},\cdots ,x_{d}\right\}$, captures the structural attributes of the vertex $v_{i}$. HOPE is designed to capture high-order proximities of symmetric transitivity in an undirected graph. In pursuit of this goal, HOPE can generate two vertex representation vectors, $U^{s}$ and $U^{t}$, both belonging to the real number space $U^{s},U^{t}\in R^{\left|V\right|\times d}$. These vectors, $U^{s}$ and $U^{t}$, are identified as source and target vectors respectively. The objective function can be delineated as follows:

(Equation 3) $$\begin{array}{c} \min_{U^{s},U^{t}}|S-U^{s}\cdot U^{t}{|}_{F}^{2} \end{array}$$

The structure of the preserved graph can be perceived as a measure of the similarity among the preserved nodes. S presents the matrix of Higher-Order Proximity.

#### Gradient boosting decision tree

Gradient boosting^25^ is an ensemble machine learning methodology that iteratively amalgamates weak 'learners' to form a powerful, singular learner. In this research, we employ a regression tree model to train a dataset consisting of known values of x and their associated yvalues. The aim is to derive an approximation function, $\left(\hat{F}\left(x\right)\right)$, that minimizes the expected value of a predetermined loss function, denoted as $F_{L}\left(y,F\left(x\right)\right)$. The approximation function is defined as follows:

(Equation 4) $$\begin{array}{c} \hat{F}=argminEX\left(F_{L}\left(y,F_{L}\left(x\right)\right)\right) \end{array}$$

In this context, y is a real value. The gradient boosting decision tree model operates under the assumption that y is a real-valued variable and aims to find an approximation in the form of a weighted sum of functions, $h_{i}\left(x\right)$ , derived from a certain class H. These functions can be referred to as weak learners, defined as follows:

(Equation 5) $$\begin{array}{c} F\left(x\right)=\sum_{i=1}^{M}\gamma_{i}h_{i}\left(x\right)+\text{const}\; \end{array}$$

Adhering to the principle of empirical risk minimization, this method seeks an approximation function $\left(\hat{F}\left(x\right)\right)$, which minimizes the average value of the loss function on the training set reducing the empirical risk. This is achieved by initiating with a model comprised a constant function, $F_{0}\left(x\right)$, and progressively expanding it in a greedy manner:

(Equation 6) $$\begin{array}{c} F_{0}\left(x\right)=\mathrm{argmin}\sum_{i=1}^{n}F_{L}\left(y_{i},\gamma \right) \end{array}$$

Assuming there are training samples, $x_{i},y_{i},i=1\ldots n$, The cumulative model obtained in the (m−1)−th round is $F_{m-1}\left(x\right)$ Then the weak learner for the m−th round can be represented as

(Equation 7) $$\begin{array}{c} F_{mi}\left(x\right)=F_{m-l}\left(x\right)+\mathrm{argmin}\left[\sum_{l=1}^{n}F_{L}\left(y_{i},F_{m,j}\left(x_{i}\right)+h_{mi}\left(x_{i}\right)\right)\right] \end{array}$$

In these equations, $h_{m}\in H$ represents a weak learner function. The task of selecting the optimal function h at each stage for an arbitrary loss function $F_{L}$ generally poses a computationally unattainable optimization problem. As a result, a simplified approach is adopted to address this issue.

The primary concept involves employing the steepest descent step to resolve this minimization conundrum. If we consider the continuous case, where H encompasses an array of arbitrary differentiable functions on R, the model would be updated based on the subsequent equations:

(Equation 8) $$\begin{array}{c} F_{m}\left(x\right)=F_{m-l}\left(x\right)-\gamma_{m}\sum_{i=1}^{n}\nabla_{F_{x-l}}F_{L}\left(y_{i},F_{m-l}\left(x_{i}\right)\right) \end{array}$$

(Equation 9) $$\begin{array}{c} \gamma_{m}=\underset{\gamma }{\mathrm{argmin}}\sum_{i=1}^{n}F_{L}\left(y_{i},F_{m-l}\left(x_{i}\right)-\gamma \nabla_{F_{m-1}}F_{L}\left(y_{i},F_{m-l}\left(x_{i}\right)\right)\right) \end{array}$$

In these formulas, the derivatives are computed with respect to the functions $F_{i}$ for i∈{1,..,m}. Conversely, in the discrete scenario, where the set H is finite, the candidate function h chosen would align closely with the gradient of $F_{L}$. The coefficient γ can then be computed using a line search on the equations. It is important to note that this strategy is heuristic in nature, and as such, does not provide an exact solution to the problem at hand, but rather an approximation.

### Quantification and statistical analysis; additional resources

In the final evaluation of the model’s performance, we employed a 5-fold cross-validation approach. The ultimate results consisted of the mean and variance of the 5-folds, with specific evaluation metrics including AUC, AUPR, MCC, ACC, and F1.

In the subsequent comparative and ablation experiments, we randomly selected 20% of the data as the test set to evaluate the model. The specific evaluation metrics included AUC, AUPR, MCC, ACC, and F1.

In the performance comparison of SPBCMI with existing models, we utilized AUC and AUPR as evaluation metrics.
